# Materials Nanoarchitectonics for Advanced Devices

**DOI:** 10.3390/ma17235918

**Published:** 2024-12-03

**Authors:** Katsuhiko Ariga

**Affiliations:** 1 Research Center for Materials Nanoarchitectonics (MANA), National Institute for Materials Science (NIMS), 1-1 Namiki, Tsukuba 305-0044, Ibaraki, Japan; ariga.katsuhiko@nims.go.jp; 2 Graduate School of Frontier Sciences, The University of Tokyo, 5-1-5 Kashiwanoha, Kashiwa 277-8561, Chiba, Japan

**Keywords:** nanoarchitectonics, advanced device, doping control of organic semiconductor, inorganic materials nanoarchitectonics, organic molecular nanoarchitectonics, structural control

## Abstract

Advances in nanotechnology have made it possible to observe and evaluate structures down to the atomic and molecular level. The next step in the development of functional materials is to apply the knowledge of nanotechnology to materials sciences. This is the role of nanoarchitectonics, which is a concept of post-nanotechnology. Nanoarchitectonics is defined as a methodology to create functional materials using nanounits such as atoms, molecules, and nanomaterials as building blocks. Nanoarchitectonics is very general and is not limited to materials or applications, and thus nanoarchitecture is applied in many fields. In particular, in the evolution from nanotechnology to nanoarchitecture, it is useful to consider the contribution of nanoarchitecture in device applications. There may be a solution to the widely recognized problem of integrating top-down and bottom-up approaches in the design of functional systems. With this in mind, this review discusses examples of nanoarchitectonics in developments of advanced devices. Some recent examples are introduced through broadly dividing them into organic molecular nanoarchitectonics and inorganic materials nanoarchitectonics. Examples of organic molecular nanoarchitecture include a variety of control structural elements, such as π-conjugated structures, chemical structures of complex ligands, steric hindrance effects, molecular stacking, isomerization and color changes due to external stimuli, selective control of redox reactions, and doping control of organic semiconductors by electron transfer reactions. Supramolecular chemical processes such as association and intercalation of organic molecules are also important in controlling device properties. The nanoarchitectonics of inorganic materials often allows for control of size, dimension, and shape, and their associated physical properties can also be controlled. In addition, there are specific groups of materials that are suitable for practical use, such as nanoparticles and graphene. Therefore, nanoarchitecture of inorganic materials also has a more practical aspect. Based on these aspects, this review finally considers the future of materials nanoarchitectonics for further advanced devices.

## 1. Introduction

The global community is confronted with a multitude of challenges, including those related to energy [1,2,3,4,5,6,7], the environment [8,9,10,11,12,13,14], medicine [15,16,17,18,19,20,21], and information [22,23,24,25,26,27,28]. The development of functional materials represents a crucial step in addressing these challenges and paving the way for a more sustainable future. It is imperative that materials be developed which are capable of meeting a multitude of demands through the utilization of a diverse array of material chemistries. In this context, it is crucial to regulate the nanostructure of functional materials. The internal nanostructure of a given material can vary significantly, resulting in notable differences in the material’s properties and functions [29,30,31,32,33]. An increase in the interfacial area and optimization of the relative arrangement of each component can result in a significant improvement in functional efficiency [34,35,36,37,38]. The advent of nanotechnology has served to reinforce the importance of these nanostructures. Advances in nanotechnology have enabled the observation of structures at the atomic and molecular levels [39,40,41,42,43]. Furthermore, the physical properties of such nanostructures and nanospaces have been elucidated [44,45,46,47,48]. The subsequent phase in the advancement of functional materials is to integrate the insights derived from nanotechnology into the material development process. In other words, it is to reconsider functional materials using nanounits, including atoms, molecules, and nanoparticles. This is the responsibility of nanoarchitectonics, which is a concept of post-nanotechnology [49]. Similarly, the concept of nanotechnology was first proposed by Richard Feynman in the mid-20th century [50,51], and nanoarchitectonics was subsequently introduced by Masakazu Aono in the early 21st century [52,53].

**Figure 1:**
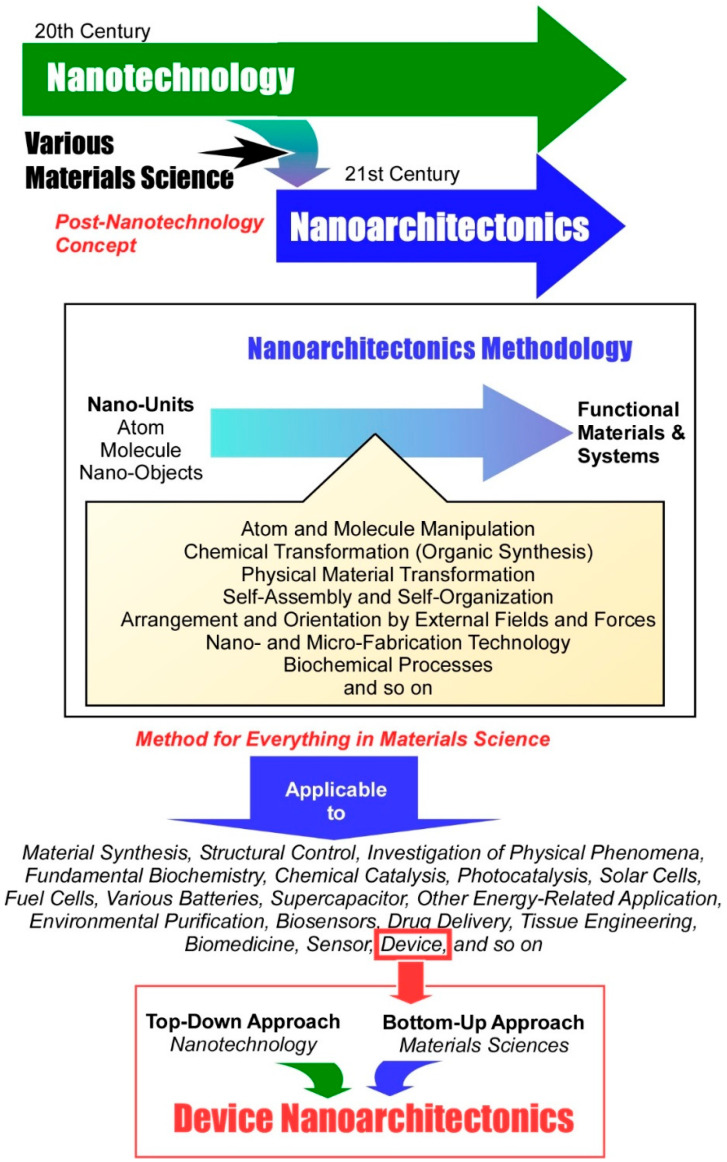
Nanoarchitectonics as the concept of constructing functional material systems from the fundamental building blocks of atoms, molecules, and nanomaterials (from the **top**) and device nanoarchitectonics as the convergence of the top-down and bottom-up approaches (**bottom**) [54,95].

Nanoarchitectonics is the concept of constructing functional material systems from the fundamental building blocks of atoms, molecules, and nanomaterials (Figure 1). In this process, a combination of atom and molecule manipulation, chemical transformation (such as organic synthesis), physical material transformation, self-assembly and self-organization, arrangement and orientation by external fields and forces, nano- and micro-fabrication technology, and biochemical processes are employed [54,55]. The creation of functional structures and the conversion of molecules into materials have also been subjects of extensive studies in past histories. The processes of self-assembly in supramolecular chemistry [56,57,58,59,60], metal–organic frameworks (MOFs) by coordination chemistry [61,62,63,64,65], covalent organic frameworks (COFs) by polymer chemistry [66,67,68,69,70], and template synthesis in materials science [71,72,73,74,75] all serve functions similar in parts to nanoarchitectonics. Furthermore, self-assembled monolayers (SAMs) [76,77,78,79,80], the Langmuir–Blodgett (LB) method [81,82,83,84,85], and layer-by-layer (LbL) assembly [86,87,88,89,90], which combine molecular assemblies and interface science technology, have also been often employed. In fact, they exhibit a pronounced nanoarchitectonics character. Therefore, nanoarchitectonics does not represent an entirely novel field of inquiry; rather, it offers an integrated conceptual framework that encompasses nanotechnology and a wide range of materials sciences [91,92].

As evidenced by the preceding background description, nanoarchitectonics is a highly general concept that can be applied without being limited to specific materials or applications. All materials are composed of atoms and molecules. Consequently, the concept of nanoarchitectonics, which constructs functional materials from units such as atoms and molecules, may be regarded as the ultimate methodology that can be applied to all materials. In analogy with the theory of everything [93], which represents the ultimate goal of physics, nanoarchitectonics may be considered a method for everything in materials science [94,95].

The increasing prevalence of nanoarchitectonics in a diverse array of fields is also evidenced by the growing number of publications that utilize the term “nanoarchitectonics” in their paper titles. The aforementioned papers span a diverse range of disciplines, encompassing material synthesis [96,97,98,99,100,101,102], structural control [103,104,105,106,107,108,109], the investigation of physical phenomena [110,111,112,113,114,115,116], fundamental biochemistry [117,118,119,120,121,122,123], chemical catalysis [124,125,126,127,128,129,130], photocatalysis [131,132,133,134,135,136,137], solar cells [138,139,140,141,142,143,144], fuel cells [145,146,147,148,149,150,151], various batteries [152,153,154,155,156,157,158], supercapacitors [159,160,161,162,163,164,165], and other energy-related applications [166,167,168,169,170,171,172]. The concept of nanoarchitectonics is also being employed in a number of practical fields, including environmental purification [173,174,175,176,177,178,179], biosensors [180,181,182,183,184,185,186], drug delivery [187,188,189,190,191,192,193], tissue engineering [194,195,196,197,198,199,200], and biomedicine [201,202,203,204,205,206,207]. Furthermore, the concept of nanoarchitectonics is being employed in the integration of artificial structures with organic, bio, and nanomaterials, as evidenced by its use in the sensor [208,209,210,211,212,213,214] and device fields [215,216,217,218,219,220,221].

Indeed, it can be argued that device technology has been a significant beneficiary of the advancements in nanotechnology. Significant progress has been made in the precise structural aspects of device development due to the advancement of various microfabrication technologies and the evaluation of nanostructures. In the context of nanoarchitectonics, it is valuable to consider the role of nanotechnology in the development of device applications. This may provide a solution to the widely recognized problem of integrating top-down and bottom-up approaches in functional system development [222,223,224]. This is because microfabrication technology, which has been a highly influential force in nanotechnology, is a representative example of a top-down approach. In contrast, nanoarchitectonics, which involves the construction of functional materials from the atomic and molecular levels, is a powerful bottom-up approach. Device nanoarchitectonics will represent the convergence of the top-down and bottom-up approaches that have been identified as being essential. It will serve as an exemplar of the convergence of nanotechnology and materials science.

In light of the aforementioned context, this review will examine a number of examples pertaining to nanoarchitectonics as it relates to devices. This review presents a selection of recent publications on devices with the term “nanoarchitectonics” in their title, in order to figure out the realistic effects of nanoarchitectonics. Therefore, the described topics do not cover all the existing science and technology. Furthermore, this review also introduces other device papers that incorporate aspects of nanoarchitectonics. The following section presents the aforementioned papers, which have been roughly categorized into two distinct groups: organic molecular nanoarchitectonics and inorganic materials nanoarchitectonics. It should be noted that this selection does not represent a comprehensive overview of all relevant examples. However, it is believed to reflect trends and characteristics. Based on these considerations, this review also contemplates the prospective evolution of materials nanoarchitectonics in the context of advanced devices.

## 2. Organic Molecular Nanoarchitectonics

The construction of these devices is based on microfabrication technology. However, the characteristics of the devices are contingent upon the materials from which they are constructed in nanoscale. The functionality of devices that exhibit optical or electronic characteristics is contingent upon the properties of the molecules that perform those functions. In other words, the development of functional molecules represents a significant challenge and potential breakthrough. If we consider the development of functional molecules as an effort to create and assemble basic molecules, it can be said that this is the result of molecular nanoarchitectonics. In particular, organic molecular nanoarchitectonics, which encompasses the design and synthesis of organic molecules, represents a significant factor in the development of devices. The following section will present a number of examples that align with this concept and will examine the key elements involved.

**Figure 2:**
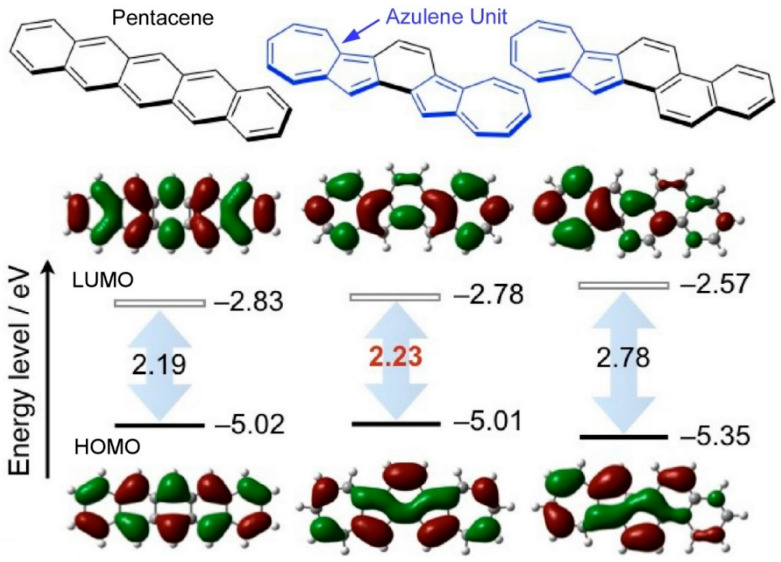
Nanoarchitectonics of introducing azulene into linear π-extended polycyclic aromatic hydrocarbons where the gap between HOMO and LUMO can be reduced to a level comparable to that of [n]acene. Reprinted with permission from [225]. Copyright 2023 Oxford University Press.

Rigid, planar carbon nanostructures with extended π-conjugation represent an attractive option for the development of nanoarchitectonics-based devices. They are notable for their distinctive properties, including high carrier mobility, robust absorption and emission in the long wavelength region, and the material properties of molecular assemblies, which are influenced by the control of intermolecular interactions in the condensed state. One of the factors that determines these functional properties is the mode and extent of π-extension. The development of functional molecules exhibiting unique photophysical and electronic properties can be achieved through the appropriate chemical and structural modifications of molecular nanoarchitectonics. Acenes have been the subject of considerable attention as a class of linearly π-extended polycyclic aromatic hydrocarbons that exhibit promising thin-film organic field-effect transistor performance. Murai, Takai, and colleagues have reported the nanoarchitectonics of introducing azulene into linear π-extended polycyclic aromatic hydrocarbons (Figure 2) [225]. Azulenes are a class of polycyclic aromatic hydrocarbons that have the potential to be utilized as linear π-extended structural isomers of pentacene and picene. New derivatives with two symmetrically fused azulene rings were synthesized in order to further elucidate the effect of incorporating the azulene ring. It was discovered that the gap between the highest occupied molecular orbital (HOMO) and the lowest unoccupied molecular orbital (LUMO) (HOMO–LUMO gap) can be reduced to a level comparable to that of [n]acene. Additionally, the researchers observed that the compounds exhibited high stability against air under visible light, with a narrow HOMO–LUMO gap comparable to that of pentacene. In accordance with this HOMO–LUMO gap, the absorption band exhibited a red shift. An X-ray single-crystal structure analysis revealed that the five fused azulene rings adopted a herringbone-packing structure, which is the result of a balance of CH–π and π–π interactions. Organic field-effect transistors were fabricated in a bottom-gate/bottom-contact configuration, utilizing a 400 nm thick SiO_2_ layer as the gate dielectric. This novel derivative comprising azulene rings was synthesized via thermal deposition under high vacuum conditions. The transfer and output curves of the thin-film organic field-effect transistors exhibited the expected behavior for a normally off field-effect transistor. The fabrication of organic field-effect transistor devices with this derivative resulted in the observation of typical p-type behavior. The results indicate that the molecular nanoarchitectonics of fusing azulene to carbon and heterocycles may be a valuable approach for designing devices with specific electronic and photophysical properties. In particular, the potential of this derivative as a new class of p-type semiconductor was clearly demonstrated.

**Figure 3:**
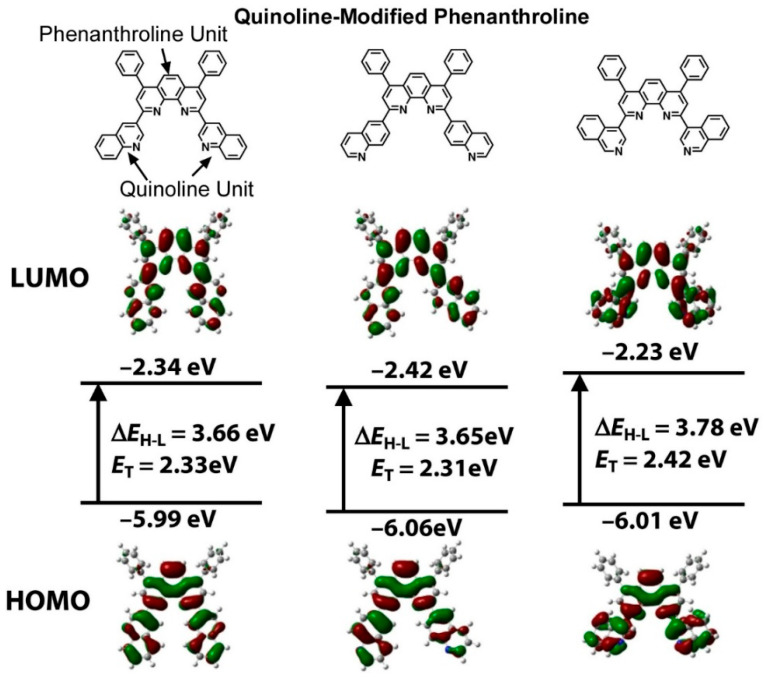
n-type exciplex host partners based on quinoline-modified phenanthroline derivatives with estimation of HOMO and LUMO, where the calculated triplet energy values were markedly larger, indicating effective confinement of triplet excitons in the emitter. Reprinted with permission from [226]. Copyright 2023 Oxford University Press.

Stable deep-red organic light-emitting devices (OLEDs) have the potential to serve as a distinctive source of illumination for plant growth and health monitoring systems. Nevertheless, the electron-to-photon conversion efficiency, expressed as external quantum efficiency, is markedly inferior to that of other primary colors. One promising strategy to enhance the external quantum efficiency of stable deep-red OLEDs is the utilization of exciplex host systems. Sasabe, Kido, and colleagues developed n-type exciplex host partners based on quinoline-modified phenanthroline derivatives [226]. The HOMO, LUMO, and triplet energy of the relevant molecules were estimated (Figure 3). The calculated triplet energy values were markedly larger, indicating the effective confinement of triplet excitons in the emitter. The developed derivatives formed exciplexes in combination with the p-type host material *N*,*N*′-di-1-naphthyl-*N*,*N*′-diphenylbenzidine, which was employed as the host material for deep-red phosphorescent OLEDs. The devices exhibited low turn-on voltages and high current density and brightness. This can be attributed to the excellent electron injection properties of these derivatives, which are caused by their higher electron affinity value. Furthermore, it demonstrates the most optimal performance among deep-red phosphorescent OLEDs. With regard to thermal stability, the material demonstrated high thermal stability with a glass transition temperature of up to 148 °C. This evidence supports the assertion that phenanthroline derivatives are promising n-type host materials. It is anticipated that this will facilitate the expeditious development and commercialization of n-type semiconductors and promote their utilization as distinctive lighting sources for plant growth and health monitoring systems.

**Figure 4:**
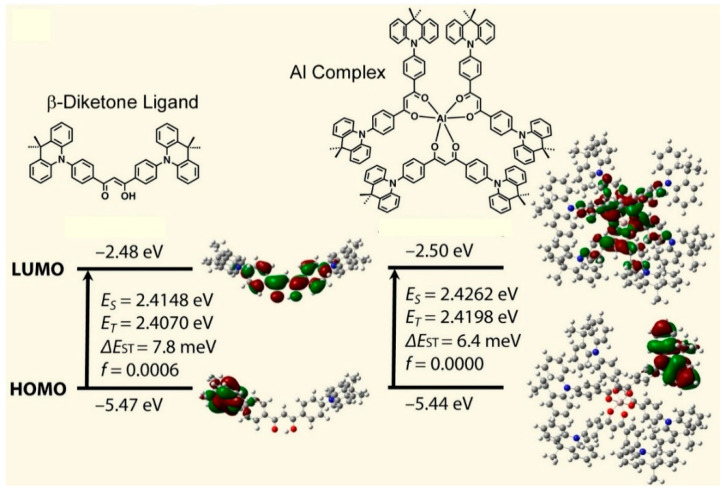
A mononuclear Al complex with a β-diketone ligand with excellent thermally activated delayed fluorescence properties. DFT calculations demonstrated that metal complexation could generate a distinctive electronic structure, which could markedly enhance the optical functions of the original diketone ligand. Reprinted with permission from [227]. Copyright 2023 Oxford University Press.

Thermally activated delayed fluorescence emitters based on widely available metal elements will emerge as the most promising contenders for the next generation of organic light-emitting diodes (OLEDs). Sasabe and colleagues developed a mononuclear Al complex with a β-diketone ligand that exhibited excellent thermally activated delayed fluorescence properties (Figure 4) [227]. In order to enhance the optical functions of the previously used molecules, molecular nanoarchitectonics was employed to modify the chemical structure of the β-diketone ligand by the addition of a donor unit. The utilization of this β-diketone derivative resulted in a notable enhancement of the photoluminescence quantum yield of the emitter, while the metal complexation led to a considerable improvement in the optical functions of the original diketone ligand in the solid state. The optical functional advantages of this complex include a very high photoluminescence quantum yield, a rapid radiative decay rate, and a short delayed fluorescence lifetime in the solid state. DFT calculations demonstrated that metal complexation could generate a distinctive electronic structure, which could markedly enhance the optical functions of the original diketone ligand. The application of nanoarchitectonics to organic light-emitting devices results in the attainment of high external quantum efficiency and low turn-on voltage, which are advantageous for the realization of low-power-consumption devices.

**Figure 5:**
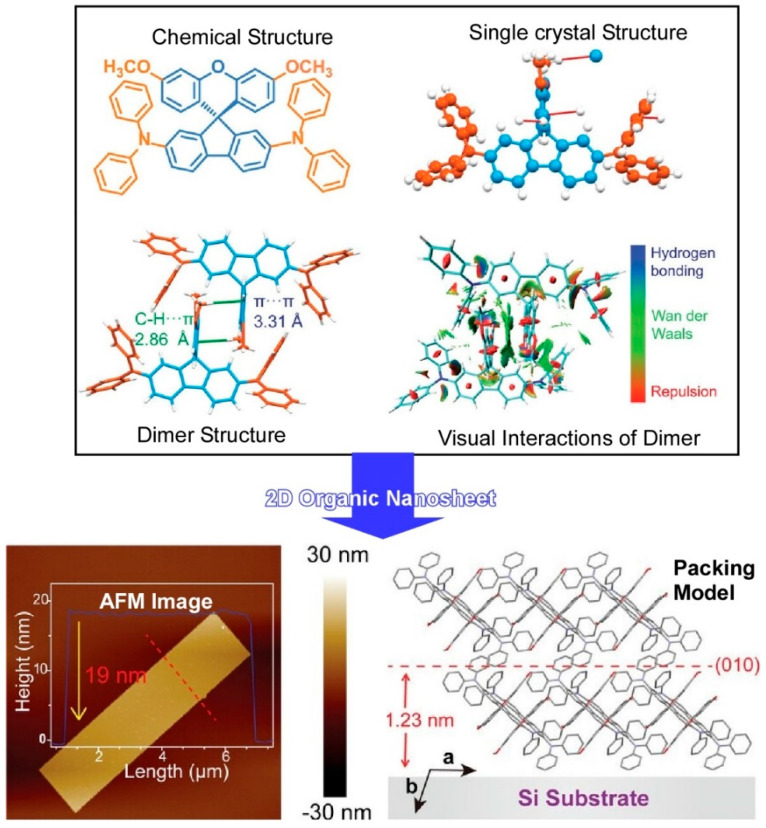
Nanoarchitectonics of ultrathin 2D organic nanosheets with denser molecular packing: (**top**) the component molecule with the orthogonal spirofluorene exanthene scaffold; (**bottom**) the formation of ultrathin 2D organic nanosheets with its AFM image and its molecular-packing model. Reprinted with permission from [228]. Copyright 2023 Wiley-VCH.

Ultrathin two-dimensional organic nanosheets exhibiting high mobility at a thickness of a few molecular layers will demonstrate enhanced device performance. In particular, the development of ultrathin 2D organic nanosheets that simultaneously exhibit high luminescence efficiency and flexibility is a highly desirable objective. In a study titled “Hierarchical nanoarchitectonics of ultrathin 2D organic nanosheets,” Zhang, Xie, and colleagues have achieved the nanoarchitectonics of ultrathin 2D organic nanosheets (thickness: 19 nm) with denser molecular packing [228]. In this component molecule, the orthogonal spirofluorene exanthene scaffold exerts an efficient steric hindrance effect on intermolecular repulsion (Figure 5). Concurrently, the methoxyl and diphenylamine groups facilitate intermolecular attraction as supramolecular segments. π-π stacking and CH···π interactions reinforce antiparallel and interpenetrating molecular packing in dimeric aggregates with proximate intermolecular distances. These molecular nanoarchitectonics are conducive to the formation of ultrathin 2D organic nanosheets. The restriction of conformational vibrations and rotations may serve to minimize non-radiative deactivation in the solid state. By employing a self-assembly method, Zhang et al. [228] have successfully fabricated ultrathin 2D organic nanosheets with a thickness of approximately 19 nm in aqueous media, despite the tight molecular packing. The ultrathin organic nanosheets can be molded into large, continuous macroscale films via a one-step drop-coating method. The organic nanosheets display sufficient flexibility. Even when the molecular stacking was denser, the ultrathin organic nanosheets prevented aggregation quenching and exhibited higher blue emission quantum yields than the amorphous films. These ultrathin 2D organic nanosheets may prove to be valuable tools for the development of flexible electrically pumped lasers and intelligent quantum tunneling systems.

**Figure 6:**
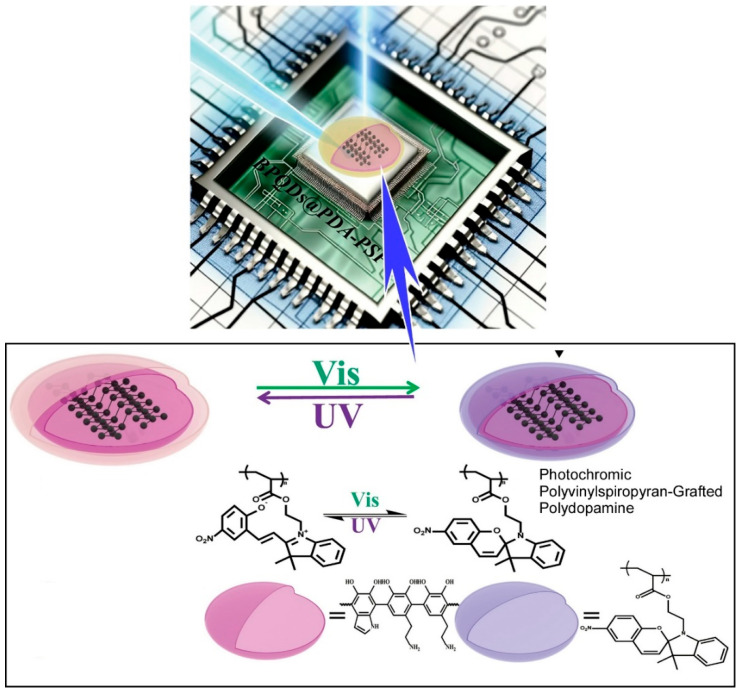
Polyvinyl spiropyran-grafted polydopamine-encapsulated structures for transient digital memristors where black phosphorus quantum dots functionalized with photochromic polyvinyl spiropyran-grafted polydopamine are employed in the construction. Reprinted with permission from [229]. Copyright 2024 Oxford University Press.

Organic/polymer resistive random-access memory (RRAM) will constitute a pivotal component in the field of bio-inspired electronics. It is anticipated that this technology will find applications in advanced information storage, intelligent perception, brain-like systems, and logic computing. Conversely, the capacity to expeditiously erase sensitive data serves to bolster both information security and intellectual property protection. He, Wang, Chen, and colleagues synthesized polyvinyl spiropyran-grafted polydopamine-encapsulated structures for transient digital memristors (Figure 6) [229]. Indeed, black phosphorus quantum dots functionalized with photochromic polyvinyl spiropyran-grafted polydopamine are employed in the construction of transient digital memristors. The film, situated between ITO electrodes, was erased rapidly by UV irradiation within six seconds. Furthermore, the film exhibited typical nonvolatile digital memristor performance when subjected to visible light irradiation. Upon UV irradiation, the closed-ring spiropyran form of the active layer is rapidly converted to the open-ring merocyanine form by “closed-to-open” isomerization, thereby enabling the information stored in the device to be rapidly and completely erased. Furthermore, the potential of this memristor for handwritten digit recognition was explored. A basic convolutional neural network comprising a convolutional layer and a pooling layer for filtering, and a fully connected layer for classification, was constructed. Following 10 epochs of training, the accuracy of digit recognition reached 96.21%.

In recent years, electrochromic devices have been employed in a multitude of applications, including those pertaining to energy conservation and display technology. Nevertheless, the advancement of lightweight, low-power, cost-effective, and environmentally benign electrochromic devices remains a pivotal objective. In their study, entitled “A facile nanoarchitectonics of electrochromic devices”, Kim, You, and colleagues developed a novel electrochromic device through the use of simple solution-cast polymerization [230]. In this instance, the researchers employed a poly(3,4-ethylenedioxythiophene) (PEDOT)/2,2,6,6-tetramethylpiperidine-1-oxy-oxidized cellulose nanofiber epoxy composite. The fabricated electrochromic device exhibited a reversible color transition between light blue (translucent state) and dark blue (colored state), dependent on the redox potential. This device is anticipated to provide a straightforward fabrication method for a range of energy-saving smart windows and high-contrast displays.

**Figure 7:**
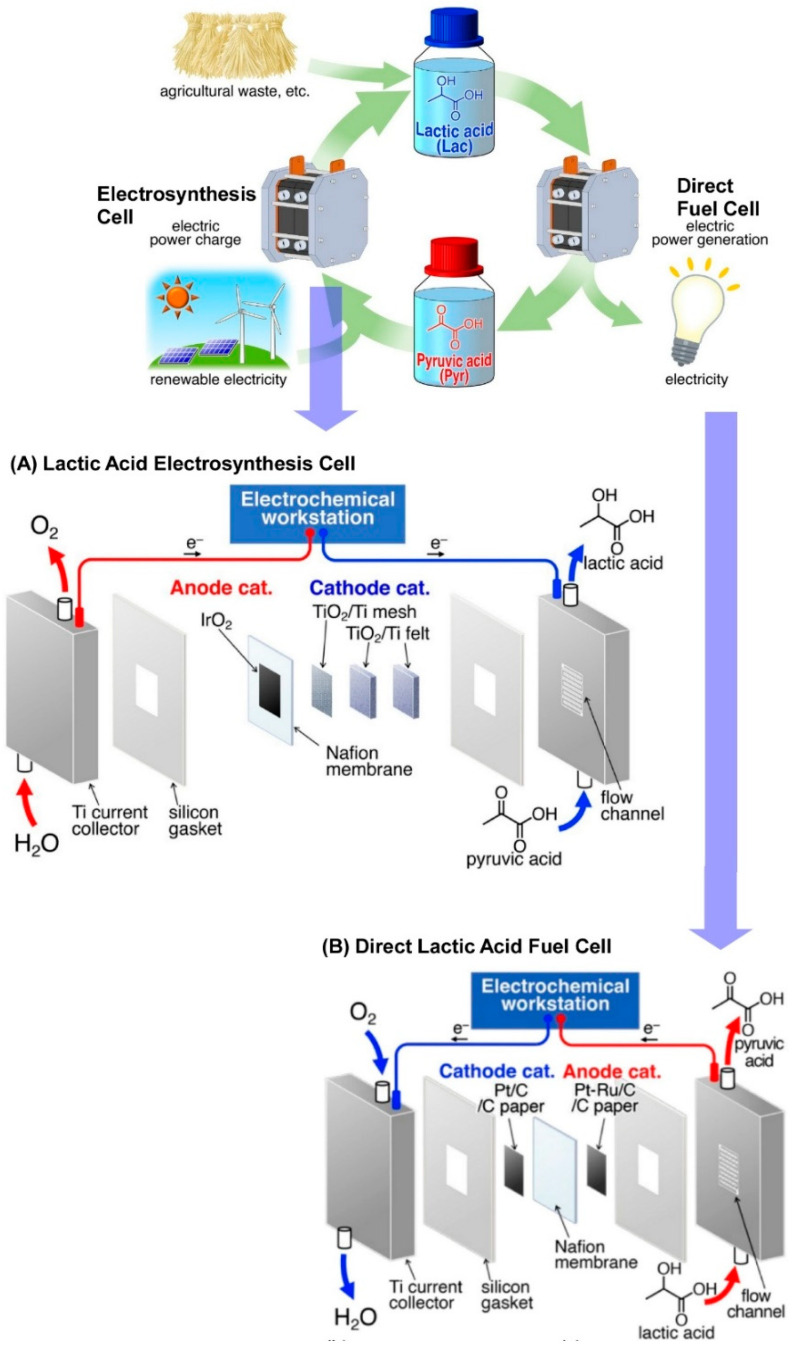
An energy cycle based on a highly selective redox reaction between lactate and pyruvate obtained from biomass resources: (**A**) a lactic acid electrosynthesis cell (LAEC); (**B**) a direct lactic acid fuel cell (DLAFC). Reprinted with permission from [231]. Copyright 2023 Oxford University Press.

The efficient storage and transport of electrical energy is a fundamental requirement for the promotion of renewable energy-based electricity. Yamauchi and colleagues have demonstrated an energy cycle based on a highly selective redox reaction between lactate and pyruvate, which are liquid at room temperature and obtained from biomass resources [231]. The objective of their system is to achieve a completely low-emission outcome. An energy storage device, namely a lactic acid electrosynthesis cell (LAEC), was constructed for the production of lactate from pyruvate. This was achieved using a membrane electrode assembly (MEA), comprising a TiO₂ cathode catalyst for the electroreduction of pyruvate and an IrOx anode catalyst for the oxidation of water (Figure 7A). The LAEC was constructed using iridium oxide nanoparticles as the anode catalyst. The LAEC exhibits complete suppression of the hydrogen evolution reaction even in highly acidic aqueous solutions. Additionally, a direct lactic acid fuel cell (DLAFC) was constructed (Figure 7B). The direct lactic acid fuel cell (DLAFC) employed platinum/cobalt and platinum–ruthenium/cobalt catalysts as the cathode and anode catalysts, respectively. The DLAFC, utilizing 1 M lactate, demonstrated a selective oxidation of lactate to pyruvate. The combination of highly selective electrochemical reactions in the LAEC and DLAFC allows for the direct storage of electrical energy in a biological liquid carrier. It is possible to complete a carbon-neutral energy cycle using the resulting energy. The LAEC/DLAFC system has the advantage of being compact with low energy consumption, as it does not require high-temperature conversion above 100 °C or the treatment of gaseous carriers.

**Figure 8:**
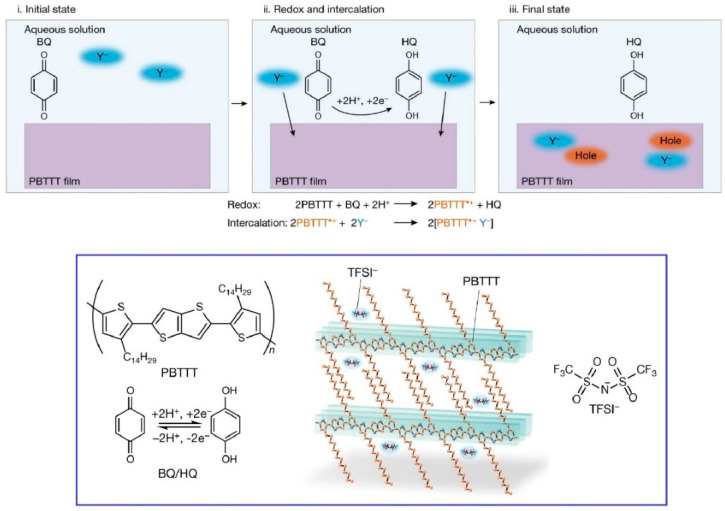
A novel coupling between proton-coupled electron transfer reactions and polymer organic semiconductors: (**top**) doping mechanism; (**bottom**) chemical structures. Reprinted with permission from [232]. Copyright 2023 Springer-Nature.

It has been proposed that ultrathin polymer organic semiconductor films have a multitude of potential applications, including the development of flexible electronic devices. Nevertheless, in comparison to single crystals of low-molecular-weight organic semiconductors, there is considerable scope for further research in the areas of fabrication and property control. One illustrative example is the control of the electronic properties of polymer organic semiconductor films by doping. Ishii, Yamashita, and colleagues have recently published a new study which demonstrates a novel coupling between proton-coupled electron transfer reactions, which are widely employed in biochemical processes and polymer organic semiconductors (Figure 8) [232]. A p-type organic semiconductor film was immersed in an aqueous solution containing a proton-coupled electron transfer reaction redox couple (benzoquinone/hydroquinone) and a hydrophobic molecular ion. The redox potential of the former can be controlled by the proton activity (pH), which is an easily manipulable parameter. The presence of p-type doping was confirmed by measuring the absorption spectrum and conductivity. The efficient doping of polymer organic semiconductor films is achieved through a synergistic reaction between proton-coupled electron transfer reactions and the insertion of hydrophobic ions. The doping level was meticulously regulated within a pH-controlled aqueous solution. In other words, the Nernst equation was employed to regulate the Fermi level of the polymeric organic semiconductor thin film through the manipulation of proton activity. This doping method is also innovative in that it can be performed in an aqueous solution at room temperature and pressure, which renders it a method that will also be useful for industrial applications. This could prove beneficial in the creation of a platform for room-temperature semiconductor processes and biomolecular electronics. It will be feasible to establish a correlation between semiconductor doping and any chemical or biochemical process that can be linked with proton activity. This method is also regarded as a promising platform for biomolecular electronics.

The diverse properties of organic molecules render them an attractive option for the creation of devices. A plethora of organic molecular structures can be synthesized through organic synthesis (molecular nanoarchitectonics). It is similarly important to consider supramolecular chemical processes, such as molecular association and intercalation, in order to control the characteristics of the devices in question. These sciences and technologies have been the subject of study in the context of coupling fields such as organic synthetic chemistry, polymer chemistry, coordination chemistry, and supramolecular chemistry with device engineering. These approaches can also be unified and interpreted as molecular nanoarchitectonics. It is anticipated that this integrated approach, which transcends the boundaries of previous fields, will further advance the field of device engineering based on organic molecules.

## 3. Inorganic Materials Nanoarchitectonics

In addition to the organic molecules previously discussed, structurally controlled inorganic materials are also useful elements for device development. The nanoarchitectonics of inorganic materials frequently permits the regulation of size, dimensions, and shape, thereby enabling the control of physical properties. This may be referred to as inorganic materials nanoarchitectonics in the context of device development. The following section will present a number of illustrative examples.

**Figure 9:**
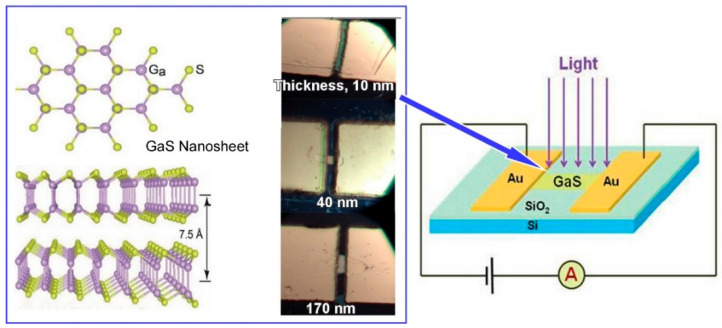
GaS nanosheet devices comprising varying thicknesses and their photoresponsive devices. Reproduced under terms of the CC-BY license [233]. Copyright 2023 MDPI.

Nanoscale solid-state devices are composed of thin sheets, typically comprising only a few atomic layers, and display remarkable electronic behavior. The electronic properties of nano solid-state devices are markedly distinct from those of conventional solid-state devices. In particular, the control of thickness is a crucial factor. Zhao, Fu, and colleagues employed an approach termed ‘thickness nanoarchitectonics’ to investigate the correlation between thickness and the Raman scattering and polarization characteristics of few-layer GaS nanosheets [233]. By means of a chemical vapor deposition method, three types of GaS nanosheets with approximate thicknesses of 10, 40, and 170 nm were produced. As the thickness of the nanosheets increased, the intensity of the Raman scattering increased at the edges of the nanosheets. Furthermore, the energy and polarization of the excitation photon had a significant impact on the edge-enhanced Raman properties. Three distinct GaS nanosheet devices, comprising varying thicknesses, were fabricated and their photocurrents were subsequently measured (Figure 9). The GaS nanosheet devices with thicknesses of 40 and 170 nm exhibited positive photoresponses, despite the photocurrents being relatively low. In contrast, the thinnest 10 nm GaS nanosheet device exhibited a substantial current even in the absence of light, despite its relatively weak response to light. Further studies demonstrated that there were differences in the spatial patterns of Raman imaging in relation to GaS thickness, excitation light wavelength, and polarization. These findings have implications for the potential applications of GaS and other transition metal sulfides in fields such as photocatalysis, electrochemical hydrogen production from water splitting, energy storage, nonlinear optics, gas sensing, photodetectors, and so forth.

**Figure 10:**
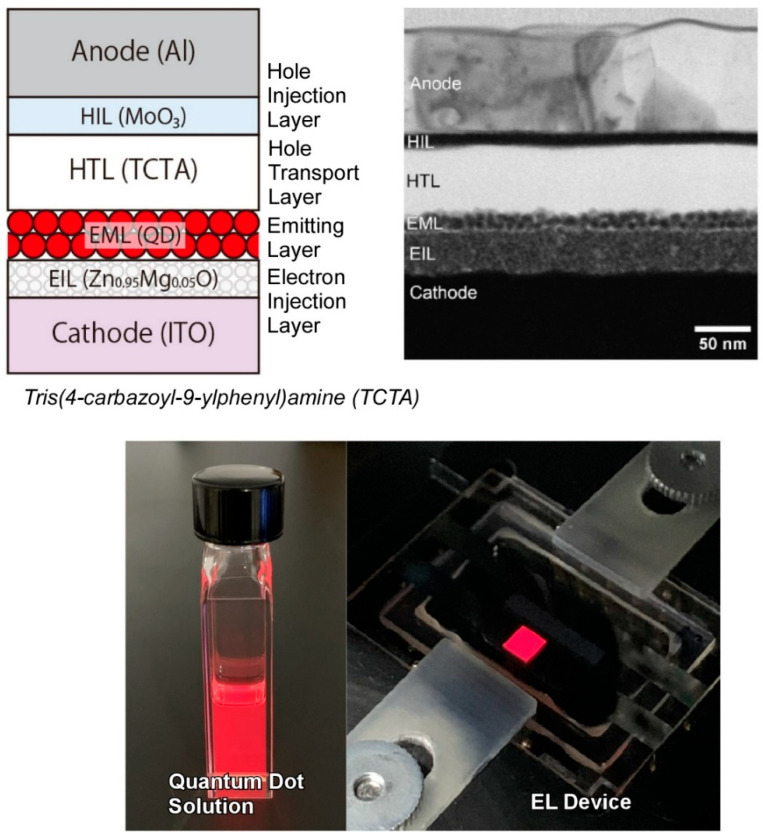
A cadmium-free red-emitting quantum dot enabled by incorporating copper into a silver indium gallium sulfide/gallium sulfide (Ag-In-Ga-S/Ga-S) core/shell quantum dot as quantum dot-LED devices. Reprinted with permission from [234]. Copyright 2023 Oxford University Press.

Colloidal quantum dots have garnered interest due to their distinctive optoelectronic characteristics, which hold promise for advancement in device engineering. Furthermore, the quantum confinement effect can be enhanced by nanoarchitectonics of the core/shell structures, thereby enabling quantum dots to be applied in light-emitting devices. Uematsu, Kuwabata, and colleagues developed a cadmium-free red-emitting quantum dot by incorporating copper into a silver indium gallium sulfide/gallium sulfide (Ag-In-Ga-S/Ga-S) core/shell quantum dot [234]. Following the application of a Ga-S shell, the quantum dots displayed a narrow red photoluminescence spectrum. The results of experiments conducted with varying Cu/Ag ratios indicate that the emission observed in these samples arises from localized carriers rather than band-edge transitions. Furthermore, the research team investigated quantum dot-LED devices (Figure 10). In this structure, the light-emitting layer is composed exclusively of Ag-Cu-In-Ga-S/Ga-S core/shell quantum dots, without the inclusion of any additional materials to facilitate charge transport. The device displayed an electroluminescence spectrum that was almost identical to the photoluminescence observed in the quantum dot solution. The core/shell quantum dot LED device exhibited high color purity red electroluminescence that met the BT2020 standard. The enhanced luminous efficiency and durability facilitate the practical utilization of the technology.

**Figure 11:**
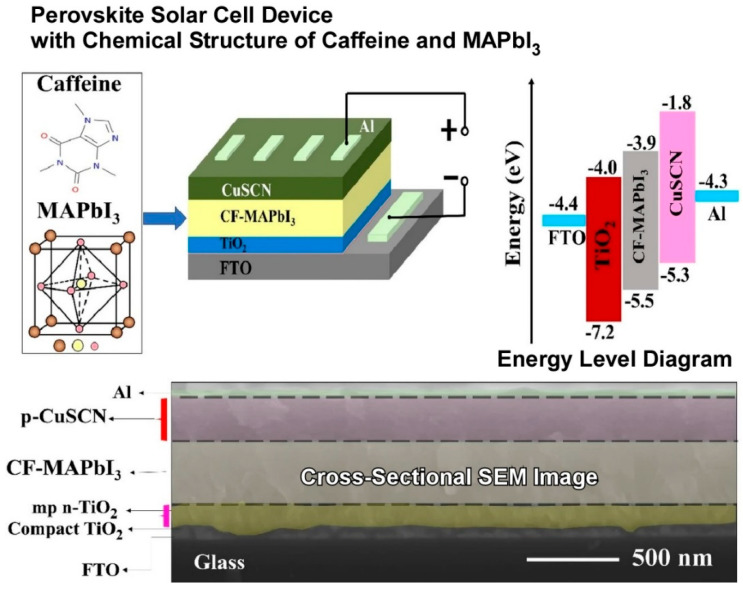
Caffeine–methylammonium lead iodide perovskite solar cell device where the introduction of caffeine into methylammonium lead iodide results in the production of a highly efficient and stable caffeine-based additive methylammonium lead iodide perovskite solar cell device. Reprinted with permission from [235]. Copyright 2023 Springer-Nature.

Nanoarchitectonics studies have been conducted in which unanticipated additives have been observed to exert control over devices. Dey and colleagues employed a caffeine additive-based nanoarchitectonics strategy, whereby caffeine (in the form of coffee powder) was introduced as a light absorber to methylammonium lead iodide, resulting in the development of a stable and efficient caffeine–methylammonium lead iodide perovskite solar cell device (Figure 11) [235]. The introduction of caffeine into methylammonium lead iodide results in the production of a highly efficient and stable caffeine-based additive methylammonium lead iodide perovskite solar cell device. The addition of caffeine to the perovskite solar cell resulted in enhanced power conversion efficiency, short-circuit current density, open-circuit voltage, fill factor, and stability when compared with the pure methylammonium lead iodide. The enhanced photovoltaic performance and stability of caffeine-added methylammonium lead iodide perovskite solar cells can be attributed to the reduction in electrical resistance and the minimization of non-radiative recombination pathways within the perovskite. The incorporation of caffeine has been demonstrated to diminish the non-radiative recombination pathways within the perovskite layer. The incorporation of caffeine into the methylammonium lead iodide light-absorbing layer has been observed to markedly enhance the electron-hole charge carriers, thereby improving the photovoltaic performance. It is anticipated that the findings will facilitate the development of large-scale industrial caffeine- or similar additive-based perovskite solar cell devices.

**Figure 12:**
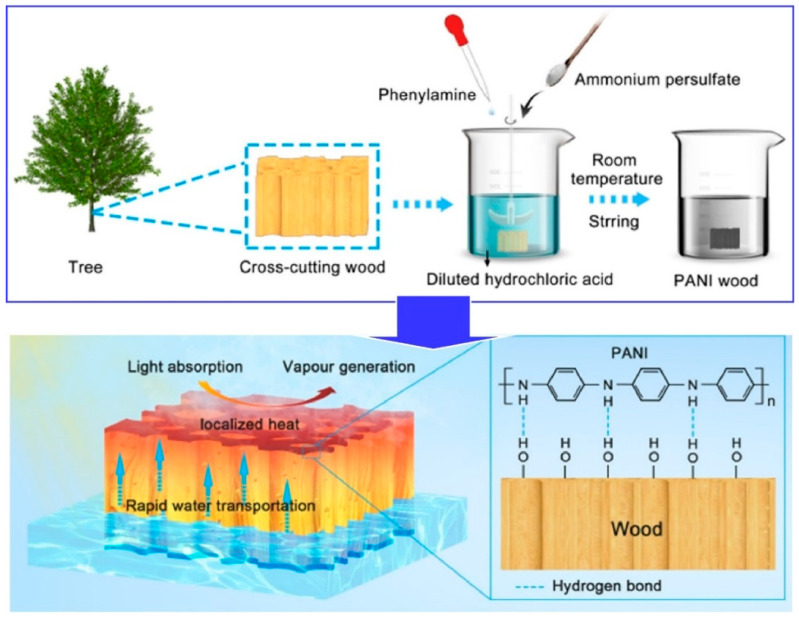
Fabricated a bilayer composite comprising uniformly incorporated polyaniline nanorods within a 3D mesoporous matrix of natural wood where the numerous aligned wood microchannels facilitate constant and rapid water transport at the air–water interface, driven by capillary forces. Reprinted with permission from [236]. Copyright 2023 Oxford University Press.

While not truly inorganic, materials such as wood are also applicable to the field of device nanoarchitectonics. The use of wood-based materials in solar steam generators has gained attention in the fields of desalination and water purification due to the cost-effectiveness and potential for renewable energy sources that these generators offer. However, it should be noted that conventional solar steam generators are not always suitable for long-term use. To this end, Li, Xu, and colleagues fabricated a bilayer composite comprising uniformly incorporated polyaniline nanorods within a 3D mesoporous matrix of natural wood, employing a one-step in situ polymerization strategy (Figure 12) [236]. The solar absorptance of polyaniline-decorated wood is exceptionally high over a broad wavelength range, due to the conjugation of coral-like polyaniline nanorods with the wood substrate. Furthermore, the intrinsic physical characteristics of wood impart to polyaniline wood a hydrophilic nature and the capacity to facilitate the transport of water. Furthermore, it displays excellent environmental and chemical resistance. The numerous aligned wood microchannels facilitate constant and rapid water transport at the air–water interface, driven by capillary forces. The polyaniline–wood composite material displays high stability and a high evaporation rate, indicating its potential as an optimal solar steam generator. The polyaniline–wood composite exhibits long-term buoyancy, which suggests that it has the potential for long-term practical application. The imminent threat of a global freshwater shortage is a direct consequence of the deterioration of global ecosystems. The generation of interfacial steam by solar means, as exemplified by this nanoarchitectonics approach to material design, has the potential to provide a solution to the global water crisis.

**Figure 13:**
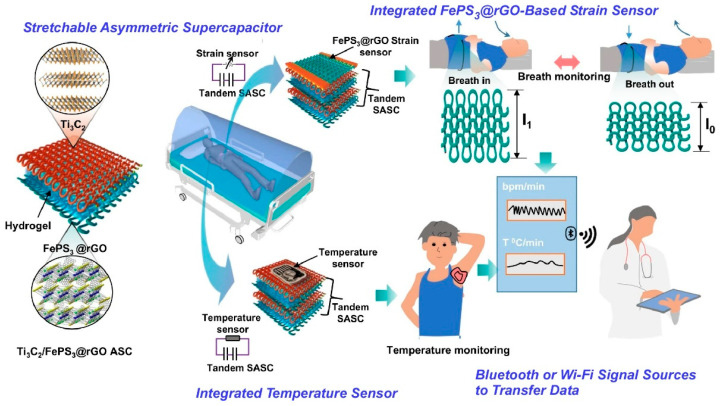
A remote health monitoring system based on a telemedicine platform for remote health assessment by an integrated nanoarchitectonics approach in which the electrodes of the stretchable asymmetric supercapacitor and strain sensor were composed of a composite of FePS_3_ and reduced graphene oxide coated on a stretchable fabric. Reproduced under terms of the CC-BY license [237]. Copyright 2022 Springer-Nature.

In light of the emergence of a number of novel infectious diseases, the necessity for remote monitoring of infected individuals has become paramount. This is particularly important in hospitals, where infected patients must be isolated to prevent the transmission of pathogens to medical personnel. It would be advantageous to develop wearable health sensor devices that are capable of monitoring patients remotely. A number of infectious diseases can be monitored for infection status through the use of various physiological indicators, including abnormal body temperature, respiratory rate, and diastolic blood pressure. As reported by Pumera and colleagues, a remote health monitoring system has been developed which employs a telemedicine platform for health assessment remotely by an integrated nanoarchitectonics approach [237]. This system incorporates a stretchable asymmetric supercapacitor as a portable power source and a sensor capable of monitoring the physical health status of humans remotely in real time (Figure 13). The system is textile-based and comprises a high-performance stretchable asymmetric supercapacitor and a strain sensor. The electrodes of the stretchable asymmetric supercapacitor and strain sensor were composed of a composite of FePS_3_ and reduced graphene oxide (rGO), which were coated on a stretchable fabric. Upon stretching the FePS_3_@rGO composite, a notable decline in the brightness of the red LED was observed, accompanied by a discernible alteration in its electrical conductivity. Strain sensing is a process whereby mechanical strain is converted into a detectable electrical signal. Furthermore, the stretchable asymmetric supercapacitor can be employed to power a temperature sensor positioned beneath the armpit, thereby facilitating the monitoring of body temperature. The transmission of data from real-time monitoring of respiration and body temperature via wireless communication to a hospital cloud system for clinical evaluation is a viable option. The system permits patients to monitor these health indicators without direct contact with medical personnel. The wireless device developed in this study would be beneficial in situations where infected patients require isolation to prevent the transmission of pathogens. Moreover, this research provides a foundation for the advancement of innovative wearable e-health monitoring systems based on flexible and stretchable energy storage devices.

Inorganic materials and their hybrid counterparts exhibit a range of distinctive properties. In comparison to organic materials, inorganic materials possess a structure that is less flexible, yet it is relatively straightforward to precisely control the structure. The field of nanoarchitectonics, which encompasses techniques such as precise thickness control and core/shell structural design, is employed in the development of devices. In comparison to organic molecular nanoarchitectonics, which is somewhat more development-oriented, inorganic materials nanoarchitectonics demonstrates greater strengths in practical application. This is presumably due to the fact that research into inorganic materials in nanostructure control has made significant advancements, resulting in the identification of specific groups of materials that are well-suited for practical applications. This characteristic will be a crucial factor in advancing device nanoarchitectonics from the research stage to practical application.

## 4. Conclusions and Future Perspectives

As previously stated in the introduction, it is crucial to consider the role of nanotechnology in the evolution towards nanoarchitectonics, particularly in relation to its impact on device hardware applications. This represents a solution to the well-known problem of combining top-down and bottom-up approaches in the development of functional systems. The microfabrication techniques that are prominent in nanotechnology are an example of a top-down approach, whereas device nanoarchitectonics, which involves the assembly of functional materials from atoms and molecules, is a powerful bottom-up approach. Device nanoarchitectonics will represent the convergence of top-down and bottom-up approaches. It will serve as an illustrative example of the convergence of nanotechnology and materials science.

In this review, components are roughly divided into organic compounds and inorganic materials. Although the fundamental parts of device functional architecture are almost the same, each has its own characteristics. For example, many devices that exhibit optical and electronic functions are heavily dependent on the properties of the molecules that perform their functions. In other words, the development of functional molecules is a major key. Organic molecules have diverse properties, and their characteristics are attractive for device creation. The structures of organic molecules are diverse, and there are various control elements such as π-conjugated structures, chemical structures of complex ligands, steric hindrance effects, molecular stacking, isomerization and color changes due to external stimuli, control of selective redox reactions, and doping control of organic semiconductors by electron transfer reactions, to name just a few. The structures of organic molecules can be created in a variety of ways through organic synthesis (molecular architectonics). In addition, supramolecular chemical processes such as molecular association and intercalation are also important for controlling device characteristics. On the other hand, nanoarchitectonics of inorganic materials often allows control of size, dimension, and shape, and the associated physical properties can also be controlled. Among the examples of structural elements given here, precise thickness control, core/shell structure, additive control, environmental and chemical resistance, and composite materials for multifunctional devices are recognized. Generally speaking, nano-inorganic materials are characterized by the ease of precise structural control. In addition, there are specific material groups suitable for practical use, such as nanoparticles and graphene. Therefore, inorganic materials nanoarchitectonics is also closer to the practical stage. Of course, detailed examinations and confirmations of actual performances and functions of the materials and devices prepared with nanoarchitectonics concepts are important. In particular, attention must be given to concrete parameters, temperature conditions, pressure conditions, and the selection of materials to ensure the reproducibility, durability, stability, effectiveness, environmental impacts, sustainability, chemical availability, and cost-benefits upon comparisons with the control matters of existing technologies, materials, and real devices with detailed statistical analyses. These investigations will practically prove true the meanings of the nanoarchitectonics approach.

In the field of device nanoarchitectonics, there are notable distinctions between organic molecules and inorganic materials. However, the fundamental methodology of constructing functional devices using nanounits is largely similar. Rather than developing these large component elements independently, it would be more beneficial to hybridize and integrate them in order to construct more functional devices. The properties of the functional materials that are to be incorporated as components are diverse and somewhat idiosyncratic. Furthermore, the anticipated functional outcome is also highly variable. Therefore, it is anticipated that a multitude of functions will be expressed within the same device. It may prove challenging for humans to process this diversity-based approach, given past experience and existing achievements. In order to develop functional devices in an efficient and innovative manner, it will be necessary to utilize the capabilities of artificial intelligence. It is evident that machine learning [238,239,240] and materials informatics [241,242,243] have made significant contributions to materials sciences and other related fields. Additionally, there are publications that address the integration of nanoarchitectonics and artificial intelligence [244,245]. In the context of device development, there is a substantial accumulation of data that can be utilized as a basis for artificial intelligence, given that the materials employed, the structure, the function, and the output are all subject to rigorous examination. The further development of device nanoarchitectonics will be contingent upon the introduction of artificial intelligence. Furthermore, there is a pressing need for the advancement of device nanoarchitectonics into the realm of practical devices. At that juncture, it will be increasingly advantageous to integrate it with microfabrication technology oriented towards mass production. This indicates that the integration of bottom-up science and top-down technology will be a highly beneficial approach. The advent of artificial intelligence will facilitate this process. Nanoarchitectonics approaches with artificial intelligence may even provide the potential for long-term practical applications without providing sufficient empirical data or tests that could substantiate durability and resilience over extended periods. In particular, such considerations have to be taken with the viewpoint that the materials used are environmentally friendly even though it lacks a life-cycle analysis or comparative assessment against conventional materials. The nanoarchitectonics approach effectively integrates devices across various applications as seen in possible examples of remote health monitoring (for the practical clinical effects see the corresponding original papers). Many other systems such as organic photoelectronic and organic photovoltaic devices are good candidates for integrated nanoarchitectonics devices that are also designed with artificial intelligences developed upon previously accumulated knowledges through detailed comparisons of performance metrics with similar materials from previous studies.
